# Evaluation of the Relationship Between Smartphone Addiction and Lower Back Pain and Neck Pain Among Medical Students at Al-Baha University

**DOI:** 10.7759/cureus.60561

**Published:** 2024-05-18

**Authors:** Hasan Ali Abdullah AlAidarous, Nawaf S Alghamdi, Rajeh S Alghamdi, Mohammed A Alghamdi, Mohammed A Alshahrani, Ahmed M Alghamdi, Eyad A Alzahrani, Khalid N Alghamdi, Naif M Alzahrani, Abdulrahman A Alghamdi

**Affiliations:** 1 Surgery, Faculty of Medicine, Al-Baha University, Al-Baha, SAU; 2 Medicine, Faculty of Medicine, Al-Baha University, Al-Baha, SAU

**Keywords:** smartphone addiction scale short version (sas-sv), lower back pain (lbp), back pain, neck pain, al-baha university, medical students, smartphone addiction

## Abstract

Introduction

In recent years, the increased use of smartphones has adversely affected students, leading to issues like musculoskeletal pain. Therefore, our objective was to assess the correlation between smartphone addiction and neck and lower back pain.

Methodology

An observational cross-sectional study was conducted at Al-Baha University, Al-Baha, Saudi Arabia. The Smartphone Addiction Scale Short Version (SAS-SV) was used to measure the level of smartphone addiction while the Nordic Musculoskeletal Questionnaire (NMQ) was utilized to evaluate musculoskeletal pain.

Results

Smartphone addiction was prevalent in 72% of the participants (n = 293). Significantly, lower back pain was associated with smartphone addiction (p-value = 0.004). However, none of the demographic characteristics were associated with neck or lower back pain (p-value > 0.05). Students in clinical years had a higher risk of neck pain than those in an internship (p-value = 0.048).

Conclusion

Almost two-thirds of the students were addicted to smartphones, with a significant association with lower back pain. Students addicted to their smartphones had a higher risk of developing lower back pain, while clinical-year students had a higher risk of developing neck pain. It’s important to raise awareness about the health and safety dangers linked to smartphones and other devices.

## Introduction

The number of mobile cellular subscriptions was reported to be 8.3 billion in 2023 [1]. While this rise in smartphone utilization improves medical education, it also negatively affects students [2-4]. One of these negative effects is musculoskeletal pain, which arises from maintaining poor postures for a long time, such as prolonged neck flexion, while looking down at the smartphone [5].

A global study found that 568 million people have lower back pain (LBP), and 223 million have neck pain [6]. Looking specifically at medical students, a study in Belgrade found that 75.8% of fourth-year medical students suffer from LBP at some point in their lives [7]. Multiple studies found a significant association between smartphone addiction and pain in the neck and lower back [8-10]. A study in Turkey found a significant association between smartphone addiction and pain in the neck, wrists/hands, and upper back. Another study in Saudi Arabia found that 59.1% of university students have neck or shoulder pain while using electronic devices [11,12]. However, we noticed a lack of similar studies among medical students at Al-Baha University.

Therefore, we aim to evaluate the relationship between smartphone addiction and pain in the neck and lower back after estimating their prevalence in the medical college of Al-Baha University. The results of this study can be used to spread awareness about the prevalence and magnitude of the problem, which in turn may result in educating students and developing preventive measures.

## Materials and methods

This was an observational cross-sectional study that aimed to evaluate the relationship between smartphone addiction and LBP and neck pain among medical students at Al-Baha University, Al-Baha, Saudi Arabia. Both male and female medical students at Al-Baha University were included in this study. Students younger than 19 years old, and those in the first year, which is considered the preparatory year, were excluded. The study was approved by the Scientific Research & Ethics Committee at Al Baha University Faculty of Medicine (approval number: REC/SUR/BU-FM/2023/48, dated June 12, 2023). The participants were fully informed and agreed to participate in the study. Confidentiality and privacy were maintained throughout the study. Data access was limited to authorized researchers only. Participants were assured that their participation was voluntary and that they had the right to withdraw from the study at any time without consequences.

Sample size

According to the Deanship of Students' Affairs of Medical College at Al Baha University, the number of medical students is 450 students. Using this information, we calculated our sample size, which was 208 participants as a minimum with a 5% margin of error, 95% confidence interval (CI), and 50% distribution rate. We calculated our sample size with Raosoft, an online sample size calculator [13].

Methods and procedures

A stratified sampling technique was used in this study in which we selected medical students from the faculty of medicine at Al-Baha University and divided them into different strata according to academic level and gender (e.g., second-year females, second-year males, third-year females, third-year males). Finally, data were collected randomly from each stratum.

Data collection

The data were collected from July 15, 2023, to August 25, 2023, using an online version of a questionnaire containing three sections. The first section was related to sociodemographic data. For the second and third sections, validated questionnaires that have been previously utilized in similar research studies: (i) the Smartphone Addiction Scale Short Version (SAS‐SV), which was developed and validated in 2013, to assess the level of smartphone addiction [14] and (ii) the Nordic Musculoskeletal Questionnaire (NMQ), which was developed and validated in 1987, to assess neck and lower back pain [15].

Statistical analysis

Data was collected and cleaned in an Excel sheet (Microsoft Corporation, Redmond, Washington, United States) and then imported to R software version 4.2.2 (R Foundation for Statistical Computing, Vienna, Austria). Categorical variables were presented in numbers and percentages. Chi-square and Fisher exact tests were used to evaluate the determinants of addiction. Logistic regression models were used to determine the predictors of addiction, neck pain, and LBP. A p-value of less than 0.05 was set as the significant level of the study.

## Results

There was a total of 293 participants and the majority (36%, n=33) fell within the 21-22 age group. Most participants were female (61%, n=173) and in their clinical years (54%, n=158). Smartphone addiction was prevalent in 72% of the participants (n=210), as shown in Table 1.

**Table 1:** Sociodemographic characteristics of the respondents (N=293)

Characteristic	n (%)
Age	
19-20 years	97 (33%)
21-22 years	106 (36%)
More than 22 years	90 (31%)
Gender	
Female	178 (61%)
Male	115 (39%)
Educational level	
Basic years	104 (35%)
Clinical years	158 (54%)
Internship	31 (11%)
Addiction level	
No addiction	83 (28%)
Addiction	210 (72%)

A total of 179 participants had pain during the past 12 months, 96 participants had pain during the past week, and 52 participants had pain during both periods. Regarding musculoskeletal pain, wrist or hand pain was the most commonly reported type over the past year (25%, n=44), while neck pain was the most common over the past week (27%, n=26). In the past year, 18% experienced neck pain (n=33), and 16% experienced LBP (n=29). However, in the past week, 27% experienced neck pain (n=26, and 10% experienced LBP (n=10). Neck and lower back pain were reported by 19.2% (n=10) and 13.5% (n=7), respectively, both in the past year and the last week, as detailed in Table 2.

**Table 2:** Prevalence of musculoskeletal pain among the respondents

The site of pain	Pain during the past 12 months (N=179), n (%)	Pain during the past week (N=96), n (%)	Pain during both periods (N=52), n (%)
Wrists/Hands	44 (25%)	20 (21%)	17 (32.7%)
Neck	33 (18%)	26 (27%)	10 (19.2%)
Lower back	29 (16%)	10 (10%)	7 (13.5%)
Upper back	26 (15%)	9 (9.4%)	4 (7.7%)
Shoulders	23 (13%)	14 (15%)	7 (13.5%)
Ankles/Feet	18 (10%)	9 (9.4%)	5 (9.6%)
Elbows	3 (1.7%)	4 (4.2%)	2 (3.8%)
Hips/Thighs	2 (1.1%)	1 (1.0%)	0 (0 %)
Knees	1 (0.6%)	3 (3.1%)	0 (0 %)

Significantly, LBP was associated with smartphone addiction (p-value = 0.004), as indicated in Table 3. However, none of the demographic characteristics were associated with neck or back pain (p-value > 0.05), as presented in Table 4.

**Table 3:** Association of demographic characteristics and site of pain with addiction *Pearson's Chi-squared test; Fisher's exact test

	Addiction status		
Characteristic	Not addicted (N = 83), n (%)	Addicted (N = 210), n (%)	p-value^*^
Age			0.10
19-20 years	23 (28%)	74 (35%)	
21-22 years	38 (46%)	68 (32%)	
More than 22 years	22 (27%)	68 (32%)	
Gender			0.97
Female	50 (60%)	128 (61%)	
Male	33 (40%)	82 (39%)	
Educational level			0.8
Basic years	28 (34%)	76 (36%)	
Clinical years	47 (57%)	111 (53%)	
Internship	8 (9.6%)	23 (11%)	
Lower back			0.004
No pain	1 (5.9%)	17 (46%)	
Pain	16 (94%)	20 (54%)	
Neck			0.5
No pain	3 (14%)	10 (23%)	
Pain	19 (86%)	34 (77%)	

**Table 4:** Association of demographic characteristics with neck or lower back pain ^*^Fisher's exact test; **Pearson's Chi-squared test, Fisher's exact test

	Neck status			Lower back status		
Characteristic	No pain (N = 13), n (%)	Pain (N = 53), n (%)	p-value^*^	No pain (N = 18), n (%)	Pain (N = 36), n (%)	p-value^**^
Age			0.95			0.94
19-20 years	4 (31%)	18 (34%)		6 (33%)	12 (33%)	
21-22 years	4 (31%)	17 (32%)		6 (33%)	11 (31%)	
More than 22 years	5 (38%)	18 (34%)		6 (33%)	13 (36%)	
Gender			0.4			0.4
Female	7 (54%)	36 (68%)		10 (56%)	16 (44%)	
Male	6 (46%)	17 (32%)		8 (44%)	20 (56%)	
Educational level			0.2			0.95
Residence years	3 (23%)	5 (9.4%)		2 (11%)	3 (8.3%)	
Basic years	6 (46%)	18 (34%)		7 (39%)	14 (39%)	
clinical years	4 (31%)	30 (57%)		9 (50%)	19 (53%)	

Students in clinical years had a higher risk of neck pain, than those in an internship (95%CI 1.04-68.5, p-value=0.048). On the other hand, addicted students had a higher risk of LBP (95%CI 0.00-0.33, p-value=0.01), as highlighted in Table 5.

**Table 5:** Predictors of neck and lower back pain among the participants

	Neck pain predictors		Lower back pain predictors	
Characteristic	OR (95% CI)	p-value	OR (95% CI)	p-value
Educational level				
Internship	—		—	
Basic years	2.05 (0.31-12.5)	0.43	0.55 (0.01-16.1)	0.74
Clinical years	7.82 (1.04-68.5)	0.048	0.57 (0.04-7.63)	0.67
Gender				
Female	—		—	
Male	0.28 (0.05-1.17)	0.091	3.23 (0.68-19.1)	0.16
Addiction status				
Not addict	—		—	
Addict	0.45 (0.08-1.87)	0.31	0.05 (0.00-0.33)	0.010
Age				
19-20 years			—	
21-22 years			0.50 (0.04-5.42)	0.57
More than 22 years			0.50 (0.02-8.11)	0.64

## Discussion

We found that almost two-thirds of the students were addicted to smartphones, with a significant association with LBP only (p-value = 0.004). Neither neck pain, nor demographic characteristics were significantly associated with smartphone addiction (p-value > 0.05). Also, our results suggest that addicted students have a high risk of developing LBP and not neck pain (p-value = 0.01).

The prevalence of smartphone addiction in our study (72%, n=210) was higher than the prevalence reported in previous studies using SAS-SV [4,8,16,17]. However, it is important to note that the studies had different populations, sample sizes, and demographic characteristics. Therefore, it is difficult to make a direct comparison of the results. Also, our study showed a significant association between smartphone addiction and LBP (p-value = 0.004), while multiple previous studies showed a significant association between smartphone addiction and other factors such as educational level and neck pain [4,11]. Furthermore, two other studies supported our finding that both neck and LBP ranked among the top three most prevalent areas of musculoskeletal pain over the course of one year [4,18]. However, over the course of one week, we found that only neck pain ranked among the top three most prevalent areas. This contrasts with the two other studies, where both neck and LBP were ranked as part of the three most common areas during the same timeframe.

Although our research has strengths, such as the large sample size and the use of well-established questionnaires, it also has some limitations. We didn't take into consideration confounding factors that may be responsible for our findings, such as the long studying hours on chairs. The result of our study is difficult to generalize because the study sample was confined to only one college in one university, unlike other studies that had a larger and more diverse population. Also, we did not take into account the impact of musculoskeletal pain on daily activities and mood, unlike other studies that didn’t neglect the impact on different aspects, such as physical activity, sleeping, and mood [7,19]. 

Finally, smartphone addiction has a negative impact on student health and our findings suggest that the prevalence of smartphone addiction is high in medical students. Therefore, it is recommended to avoid excessive use of smartphones. Raising awareness about the health and safety dangers linked to smartphones and other devices should be initiated by both non-governmental and governmental organizations. For the future, we recommend conducting a longitudinal study including a larger, more diverse population.

## Conclusions

Overall, 72% of students were found to be addicted to smartphones, and they mainly suffered from LBP. Notably, the clinical-year students had the highest risk for neck pain, while addicted students had the highest risk for lower back issues. It’s crucial to spread the word about the health risks tied to smartphones and conduct a more extensive study involving diverse groups for a better understanding.

## References

[1] (2023). Ericsson Mobility Report: Q2 2023 Update.

[2] Latif MZ, Hussain I, Saeed R, Qureshi MA, Maqsood U (2019). Use of smart phones and social media in medical education: trends, advantages, challenges and barriers. Acta Inform Med.

[3] Alosaimi FD, Alyahya H, Alshahwan H, Al Mahyijari N, Shaik SA (2016). Smartphone addiction among university students in Riyadh, Saudi Arabia. Saudi Med J.

[4] Alsalameh AM, Harisi MJ, Alduayji MA, Almutham AA, Mahmood FM (2019). Evaluating the relationship between smartphone addiction/overuse and musculoskeletal pain among medical students at Qassim University. J Family Med Prim Care.

[5] Mahmoud NA, Abu Raddaha AH, Zaghamir DE (2022). Impact of digital device use on neck and low back pain intensity among nursing students at a Saudi government university: a cross-sectional study. Healthcare (Basel).

[6] Cieza A, Causey K, Kamenov K, Hanson SW, Chatterji S, Vos T (2021). Global estimates of the need for rehabilitation based on the Global Burden of Disease Study 2019: a systematic analysis for the Global Burden of Disease Study 2019. Lancet.

[7] Vujcic I, Stojilovic N, Dubljanin E, Ladjevic N, Ladjevic I, Sipetic-Grujicic S (2018). Low back pain among medical students in Belgrade (Serbia): a cross-sectional study. Pain Res Manag.

[8] Derakhshanrad N, Yekaninejad MS, Mehrdad R, Saberi H (2021). Neck pain associated with smartphone overuse: cross-sectional report of a cohort study among office workers. Eur Spine J.

[9] Mokhtarinia HR, Torkamani MH, Farmani O, Biglarian A, Gabel CP (2022). Smartphone addiction in children: patterns of use and musculoskeletal discomfort during the COVID-19 pandemic in Iran. BMC Pediatr.

[10] Alzaid AN, Alshadokhi OA, Alnasyan AY, AlTowairqi MY, Alotaibi TM, Aldossary FH (20181). The prevalence of neck pain and the relationship between prolonged use of electronic devices and neck pain in: a Saudi Arabia, cross-sectional study in Saudi Arabia. Egypt J Hosp Med.

[11] Mustafaoglu R, Yasaci Z, Zirek E, Griffiths MD, Ozdincler AR (2021). The relationship between smartphone addiction and musculoskeletal pain prevalence among young population: a cross-sectional study. Korean J Pain.

[12] Elsiddig AI, Altalhi IA, Althobaiti ME, Alwethainani MT, Alzahrani AM (2022). Prevalence of neck and shoulder pain among Saudi universities' students who are using smartphones and computers. J Family Med Prim Care.

[13] Raosoft Inc. (2004 (2024). RaoSoft® sample size calculator. http://www.raosoft.com/samplesize.html.

[14] Kwon M, Kim DJ, Cho H, Yang S (2013). The smartphone addiction scale: development and validation of a short version for adolescents. PLoS One.

[15] Kuorinka I, Jonsson B, Kilbom A (1987). Standardised Nordic questionnaires for the analysis of musculoskeletal symptoms. Appl Ergon.

[16] Alhazmi AA, Alzahrani SH, Baig M, Salawati EM, Alkatheri A (2018). Prevalence and factors associated with smartphone addiction among medical students at King Abdulaziz University, Jeddah. Pak J Med Sci.

[17] Sirajudeen MS, Alzhrani M, Alanazi A (2022). Prevalence of upper limb musculoskeletal disorders and their association with smartphone addiction and smartphone usage among university students in the kingdom of Saudi Arabia during the COVID-19 pandemic-a cross-sectional study. Healthcare (Basel).

[18] Felimban R, Alanasri S, Hawsawi N, Albazli K (2017). Musculoskeletal pain among Saudi medical students in Makkah. Int J Adv Res.

[19] Kędra A, Kolwicz-Gańko A, Sitarski D, Ewertowska P, Czaprowski D (2016). Low back pain and everyday functioning of students. Ortop Traumatol Rehabil.

